# Implementation strategies for procedural sedation and analgesia in the emergency department

**DOI:** 10.1186/s12245-017-0130-2

**Published:** 2017-02-03

**Authors:** Maybritt I. Kuypers, Frans B. Plötz, Francis Mencl

**Affiliations:** 1 Tergooi Hospital, Hilversum, The Netherlands; 2 Academic Medical Centre, Amsterdam, The Netherlands; 3 Department of Pediatrics, Tergooi Hospital, Blaricum, The Netherlands; 4 Department of Emergency Medicine, Summa Akron City Hospital, Northeastern Ohio Medical University, Akron, USA

**Keywords:** Procedural sedation, Analgesia, Emergency

## Abstract

Implementing procedural sedation and analgesia in the emergency department is still is a challenge on an international scale. Here, we describe the Dutch setting of emergency medicine and explain the strategies that were successful for the implementation of safe and effective procedural sedation and analgesia by emergency physicians. We describe strategies on how to bridge the gap of knowledge and skills and how to deal with a resistance to change.

## Correspondence

Dear Editor,

Implementing procedural sedation and analgesia (PSA) in the emergency department (ED) where emergency medicine (EM) is at an early stage of development is still a challenge on an international scale [1]. Change is frequently met with resistance and a chorus of concern. Additionally, resistance to change is often not the only challenge. Since emergency physicians (EP) are “the new kids on the block,” there are often no role models in the ED who can teach the specific skills of emergency PSA. We hope insight in our Dutch implementation strategies will serve as an inspiration and will help make “brutacaine” something of the past, on a global scale.

### Development of EM in The Netherlands: the Dutch setting

Before the introduction of emergency medicine, most physicians working in the ED were young recently graduated doctors who were marking time to get into a residency program. These individuals lacked the training and skills to provide PSA. Consequently, patients frequently had to endure treatment without adequate analgesia, the so called “brutacaine” approach, received no or insufficient sedation, or had to wait until anesthesiologists would be able to provide anesthesia in the operating room or ward.

In The Netherlands, EM is still an evolving medical specialty [2, 3]. The first 3-year EM training program commenced in 2000, but it was not until 2008 when the Dutch Medical Specialist Registration Board first recognized the EM training programs. By 2009, there were 120 certified EPs and 160 EM residents. The majority of the EPs, who completed their 3-year training program before 2008, received a retrograde certification. Currently, there are 463 EPs and there are a total of 95 hospitals in the country of which 91 have an ED. Consequently, there are still too few EPs to service all EDs around the clock, but the gap is closing. By 2011, the European Union of Medical Specialists acknowledged EM as a medical specialty. The Netherlands Society of Emergency Physicians (NSEP) is currently working hard to obtain the medical specialist status for EPs in The Netherlands, to ensure further emancipation.

Since the introduction of EPs in the ED, emergency PSA has been on the rise, first in the adult and consecutively in the pediatric population [4, 5]. This was not achieved easily, but it was clear that providing PSA would benefit ED patients and reduce delays in care as well as unnecessary admissions. Here, we describe some strategies that were successful for the implementation of PSA by EPs in The Netherlands. First, we describe strategies on how to bridge the gap of knowledge and skills and second how to deal with a resistance to change.

### Bridging the gap of knowledge and skills

In the early years, Dutch EPs in training studied and learned from textbooks, journals, or courses abroad, some EPs in training even went abroad through exchange programs or worked at a foreign ED for some period of time. These individuals saw firsthand the benefits of PSA; however, they needed a catalyst. Ultimately, one of the most important factors aiding the implementation of PSA by EPs in ED was that several training programs had invited EM consultants from other countries where PSA had become more common practice like the USA and Australia. Over a period of 6 years, these foreign EPs taught the procedures and encouraged the Dutch EPs to develop PSA in a manner that met the needs of their patients and system. When PSA was introduced, it was done with a strict and careful adherence to protocol including good monitoring of the patients and record keeping [4]. Limits were established, and potentially difficult cases were consulted with an anesthesiologist. This collaboration and cross-fertilization quickly revealed its spin-off. The ED nursing staff embraced emergency PSA as they saw the patients benefiting from this. We carefully explained what we were doing and why and we trained them to the extent they felt comfortable with their role during the PSA procedure. Patients with earlier experiences of painful reductions were visibly satisfied with this new development. Family members appreciated the more humane and faster care their children received. Other consultants soon saw the advantage of having this available for their patients in the ED 24/7. The anesthesiologists too were reassured that the EPs were cognizant of their limits and would not exclude them to the detriment of our patients. Having a physician who is experienced and confident in the procedure has two advantages: the foreign EP has immediate credibility in the eyes of the nursing staff and medical specialists, and it allowed residents and young attendings to do hands-on care under supervision and with immediate feedback, in their own setting. Something they would not be able to do if doing the training abroad. In another pioneering hospital, the anesthesia consultants were very much in favor of EPs sedating patients in the ED because they were aware of the advantages. In these hospitals, the anesthesiologists with an emergency medicine mind-set thought the first generation of EPs how to do this. This demonstrates the importance of being aware of your local allies and culture in order to see what fits best in your particular situation. At last, we can highly recommend having a solid evidence-based protocol in place and keeping close track of sedation-related adverse events. Reviewing the registration forms and discussing potential omissions and adverse events on a regular basis sets the standard for a healthy and safe learning culture. Currently, we are bridging the final knowledge gap in pediatric PSA by introducing an NSEP course specifically on this topic for both EPs and EPs in their final year of training.

### Dealing with a resistance to change

In the Netherlands, as in many countries where EM strives for recognition as a medical specialty, pioneering EPs face resistance, from established medical specialties or individual medical specialists. Frequently, it is one or a combination of several factors: fear of loss of income, fear of increased workload, fear of an erosion of power, or simply fear of the unknown. For example, not knowing what the exact competencies of the EPs are, which may consequently lead to an ungrounded concern that patient care may suffer as a result.

Sometimes, change is easiest when commenced on a local level. Either way, one first of all needs to determine the need for a certain change and the evidence base for it; next, identify the resources and training needed; and finally, look for potential obstacles and allies in advance and ensure that your providers are competent in the procedure or comfortable with the change. It may be a useful tactic not to raise a lot of attention at an early stage as this may create an opportunity for others to prepare objections and obstructions. But once things are up and running, it is important to celebrate successes, as this provides good PR and it encourages others to take part.

Following some local successes with PSA, NSEP has made it one of their top priorities to ensure the safe and effective practice of PSA in the ED by all EPs in the Netherlands. Several actions were taken to accomplish this. First, NSEP created a PSA section. One of the tasks of the PSA section was to make sure emergency PSA and EPs were on the radar of policymakers, to create awareness on the inefficient and costly ways of treating emergency patients. EPs could play an important role in the safe and efficient changes hereof. Second, the section ensured EPs were represented in the national guideline committee on PSA by non-anesthesiologists. A national guideline with a section on emergency PSA and an EM practice guideline on PSA were published and put into practice by 2013 as a result of this action [6, 7]. Third, the PSA section distributed a standard data collection form, which aided standardization for quality measurement and multi-center research, and following NSEP made adverse event registration due to PSA mandatory on a national scale. At last, to further ensure standard of practice, the PSA section introduced a specific emergency PSA course for practicing EPs and residents alike. PSA training and examination has finally become a mandatory part of EM core-curriculum and competency list as of 2015.

In conclusion, we presented several examples, which may aid the implementation of PSA in the ED setting. First, we shared our strategy on how we were able to close the knowledge and skill gap. A combination of autodidactic teaching, getting support from colleagues in house or from other nations, provision of PSA training by the national emergency medicine society, and making it a mandatory part of EM core-curriculum showed to be the keys to success in our country. Second, we provided insights on how to recognize and deal with a resistance to change. On a local level, EPs must make sure they understand who their local allies and foes are and understand what potential objections others might have, in order to be one step ahead in case a protest may rise. On a national level, EPs need to be part of national PSA guideline committees and sit at the table with policymakers as this creates awareness of the capability of the EPs and empowers them. The national EM society should distribute comprehensive protocols and registration forms, making it easier for local EDs to implement and guaranteeing a high-quality standard of PSA. Finally, we promote publications of PSA performance as this gives insight in quality and safety, continues the circle of improvement, and provides a positive image that may encourage others.

## Abbreviations

ED: Emergency department
EM: Emergency medicine
EP: Emergency physician
NVSHA: The Netherlands Society of Emergency Physicians
PSA: Procedural sedation and analgesia
